# A Randomized Controlled Pilot Intervention Study of a Mindfulness-Based Self-Leadership Training (MBSLT) on Stress and Performance

**DOI:** 10.1007/s12671-017-0715-0

**Published:** 2017-04-28

**Authors:** Juliane Sampl, Thomas Maran, Marco R. Furtner

**Affiliations:** 1 Department of Psychology, Leopold-Franzens University of Innsbruck, Innrain 52f, 6020 Innsbruck, Austria; 2 Department of Educational Science, Alpen-Adria-University of Klagenfurt, Universitätsstraße 65-67, 9020 Klagenfurt, Austria

**Keywords:** Mindfulness, Self-leadership, Academic performance, Stress, Test anxiety, Mindfulness-based self-leadership training, MBSLT, RCT intervention

## Abstract

The present randomized pilot intervention study examines the effects of a mindfulness-based self-leadership training (MBSLT) specifically developed for academic achievement situations. Both mindfulness and self-leadership have a strong self-regulatory focus and are helpful in terms of stress resilience and performance enhancements. Based on several theoretical points of contact and a specific interplay between mindfulness and self-leadership, the authors developed an innovative intervention program that improves mood as well as performance in a real academic setting. The intervention was conducted as a randomized controlled study over 10 weeks. The purpose was to analyze the effects on perceived stress, test anxiety, academic self-efficacy, and the performance of students by comparing an intervention and control group (*n* = 109). Findings demonstrated significant effects on mindfulness, self-leadership, academic self-efficacy, and academic performance improvements in the intervention group. Results showed that the intervention group reached significantly better grade point averages than the control group. Moreover, the MBSLT over time led to a reduction of test anxiety in the intervention group compared to the control group. Furthermore, while participants of the control group showed an increase in stress over time, participants of the intervention group maintained constant stress levels over time. The combination of mindfulness and self-leadership addressed both positive effects on moods and on objective academic performance. The effects demonstrate the great potential of combining mindfulness with self-leadership to develop a healthy self-regulatory way of attaining achievement-related goals and succeeding in high-stress academic environments.

## Introduction

High-performance demands at a university lead to an increase in depression, anxiety, and perceived stress of students and a higher overall rate of mental illness compared to nonuniversity peers (Bewick et al. 2010; Regehr et al. 2013). First year students seem to be especially vulnerable as they enter university at a demanding age, trying to find their social, financial, and geographical footing with a need for reorganization of their lives (Bewick et al. 2010; Lynch et al. 2011). Mindfulness is one of the techniques used for the prevention and treatment of stress (Kabat-Zinn 1990). Thus, empirical evidence shows domain-specific effects of mindfulness competencies on test anxiety (Cunha and Paiva 2012) and academic stress (Gallego et al. 2014).

Several mindfulness-based intervention studies suggest using the mindfulness practice over a fixed period of time to improve health-related issues of students (Ratanasiripong et al. 2015). As students are confronted with the demand for good grades in order to finish their studies within the allowed time span and to improve their employability academic performance plays a fundamental role in their lives. However, the stress of taking exams influences academic performance negatively (Stewart et al. 1999; Struthers et al. 2000). Furthermore, unanticipated situations and academic setbacks result in stress (Carver and Scheier 1994), depression (Ang and Huan 2006), and helplessness (McKean 1994). This demonstrates that students need to handle many challenges in order to achieve their educational goals. Research shows that a domain-specific coping style may influence the management of stressful events as well as academic performance (Struthers et al. 2000). To cope with growing demands, students need to have both stress management skills to find a healthy way of self-regulation and the competency to focus on exams in order to improve academic performance in a self-determined way.

The self-leadership approach, an extension of self-management (Manz and Sims 1980; Manz 1986), suggests the usage of specific cognitive and behavior-focused strategies to enhance achievement orientation and performance (Neck and Manz 2013). Self-leadership thereby is a self-influencing process that answers the following questions along the way to achieve specific goals: “what has to be done,” “how it has to be done,” and “why it has to be done” (Manz 1991). Furtner et al. (2012) introduced self-leadership training to the academic context and found out that self-leadership is a learnable skill. However, the effects on specifically academic outcomes remain empirically unproven in the academic domain. There is little research today that focuses on both stress prevention and performance enhancement of students (but see Hall 1999; Zenner et al. 2014). However, research discusses the specific capacity that is needed to successfully withstand negative consequences of academic pressure situations, such as suggesting a domain-specific coping style (Struthers et al. 2000).

Mindfulness, defined “as intentional and nonjudgmental awareness” and “paying attention in a particular way: on purpose, in the present moment and nonjudgmentally” (Kabat-Zinn 1994, p.4), has been of great interest in psychological research. The main focus of most studies lies on the evaluation of mindfulness-based intervention programs dealing with mental health issues in the clinical setting (Shapiro et al. 2006), such as the mindfulness-based stress reduction program (Kabat-Zinn 1990). However, independent of the research context, empirical evidence indicates that mindfulness is connected to well-being and mental health, particularly to stress, depression, and anxiety (e.g., Gallego et al. 2014; Keng et al. 2011). Thus, a meta-analysis of a variety of nonclinical studies demonstrates that mindfulness has been successful in helping healthy people to reduce stress (Chiesa and Serretti 2009). Mindfulness-based coping interventions developed for students show significant changes for trained students in terms of perceived academic stress and anxiety along with an increase in mindfulness (Brown and Ryan 2003; Howell et al. 2008). Considerable research in the academic examination domain shows that mindfulness is associated with lower trait and state anxiety, as well as lower strain during exam periods (Shapiro et al. 1998). According to these findings, mindfulness contributes to the prevention of anxiety caused by exams (Cunha and Paiva 2012) and helps students to cope better with modern student life (Lynch et al. 2011). In addition, Zenner et al. (2014) showed in their meta-analysis that mindfulness-based interventions in students and schoolchildren lead to both an improvement in stress resilience as well as in cognitive performance.

Mindfulness consists of two components: self-regulation of attention and the way in which one faces experiences. The self-regulation of attention includes a nonjudgmental observation and awareness of sensations and thoughts. It requires the ability to focus on one experience, and on the other hand, it facilitates the ability to intentionally switch from one aspect to another flexibly (Bishop et al. 2004). Both aspects are crucial for university students with regard to learning and taking exams, as they have an important role in the improvement of concentration, mind-wandering, cognitive flexibility, and memory (Alexander et al. 1989; Mrazek et al. 2013). The kind of attitude that one holds towards one’s experience is the second component of mindfulness. Facing experiences with openness and acceptance (Bishop et al. 2004) is important for coping with stressful academic situations (Zenner et al. 2014). To sum it up, mindfulness plays an important role in self-regulatory processes, influences mood (Brown and Ryan 2003), and fosters self-efficacy (Phang et al. 2015), cognitive enhancement, and performance (Zenner et al. 2014). Mindfulness, via its impact on attention and awareness (Teper et al. 2013), promotes effective executive control and further fosters the capacity for effective regulation of emotions.

Executive control is crucial for academic success (Hofmann et al. 2012). Consequently, research shows that the relation between mindfulness and achievement-related emotions is mediated by a healthy achievement-related self-regulation (Howell and Buro 2011). This healthy achievement self-regulative process can be described as an accurate and attentive targeting, observing and adapting of one’s goals (Neck and Houghton 2006). Moreover, a healthy self-regulative behavior is connected with better handling of pressure situations, stressful academic events, and performance (Struthers et al. 2000). However, students are more likely to feel stressed and experience falls in performance if they have the impression that they might not be able to improve future performances (Aspinwall and Taylor 1992). Therefore, to prevent stress, a successful intervention has to include self-determined goal setting strategies treating goals on a challenging but achievable level. Empirical research has further supported the role of setting one’s own goals to promote higher task performance (Locke and Latham 1990, 2002). The self-leadership approach claims that individuals can set specific challenging but achievable goals to foster motivation and to enhance individual performance (Neck and Manz 2013).

Self-leadership that is defined as a “process of influencing oneself” (Neck and Manz 2013, p.5) was established as an enhancement of self-management concepts (Manz and Sims 1980; Manz 1986). Empirical investigations showed that self-leadership is a unique and separate concept; however, it is related to motivational constructs such as self-regulation, self-efficacy, and intrinsic motivation (Furtner et al. 2015). Especially, goal setting and self-observation, two facets of self-regulative processes (Baumeister and Vohs 2007), are conceptually linked to several self-leadership strategies (Furtner et al. 2015). Neck and Houghton (2006) defined three main dimensions of self-leadership: (1) behavior-focused strategies, including self-goal setting, self-reward, self-punishment, self-observation, and self-cueing, (2) constructive thought pattern strategies which consist of visualizing, self-talk, and evaluating beliefs and assumptions, and (3) natural reward strategies focusing on intrinsic motivation.

The effects of self-leadership have been investigated theoretically and empirically—mainly in the occupational context (Konradt et al. 2009). This research suggests the combination of several self-leadership strategies to enhance performance, self-efficacy, and achievement motivation (Furtner et al. 2012; Neck and Manz 2013). Considering the self-leadership research about academically relevant aspects, findings show that the effects of self-leadership on academic performance are moderated by self-efficacy (Konradt et al. 2009; Prussia et al. 1998). Presently, five self-leadership intervention studies exist. They indicate that self-leadership is a trainable skill: An online-conducted self-leadership training in the occupational context of Unsworth and Mason (2012) showed that self-leadership training reduces strain and work pressure. Additionally, this intervention enhanced positive emotions and self-efficacy. Increases in self-efficacy, positive emotions, cognitive performance, and reductions in negative emotions were found by Neck and Manz (1996) who conducted a thought self-leadership intervention in the work environment of an airline. A study of Lucke and Furtner (2015) examined the effects of self-leadership training in the military context and showed that self-leadership reduced strain and improved cognitive and physical performance of soldiers. Stewart et al. (1996) could not confirm these effects; however, their study showed that people with little conscientiousness profited the most from the self-leadership intervention. Until now, little attention of self-leadership studies has been paid to the academic achievement context. Furtner et al. (2012) introduced, for the first time, a full-range self-leadership training in the academic context and proved that all self-leadership strategies can be optimized by training. However, the effects and outcomes of the self-leadership training in the academic achievement domain have not been empirically examined yet.

The theoretical connection between mindfulness and self-leadership is based on self-regulation processes (Brown and Ryan 2003; Furtner et al. 2017). Several self-regulation theories discuss the role of attention regulation regarding physical health and mental states (Baumeister et al. 1994; Carver and Scheier 1981). Carver and Scheier (1981, 2001) postulated in their control theory that individuals regulate their behavior according to discrepancies developed by a target-actual comparison. Mindfulness (Brown et al. 2007; MacKenzie and Baumeister 2015) and self-leadership (Neck and Houghton 2006) are connected with regulative processes consisting mainly of self-observation and goal setting. Thus, being mindful is always in relation to a goal. For example, to be aware in the present moment is a goal in itself. Thereby, the self-regulatory observation component is needed, as it regulates the awareness to focus on this target (Lutz et al. 2008; Tang et al. 2007). The same mechanisms play a crucial role in self-leadership; in order to increase one’s personal effectiveness and achievement, one needs a goal. During the goal attainment process, the regulatory component of self-observation processes is needed. During self-observation, feedback concerning the achievement process is given. This process facilitates a refocusing on the goal after any target deviation (Neck and Houghton 2006). Additionally, a better awareness promoted by mindfulness supports flexible and adaptive responses to events and helps to reduce automatic or impulsive reactions (Brown and Ryan 2003; Ryan and Deci 2004). In accordance with these findings, Furtner et al. (2017) showed that mindful observing is related to self-leadership.

However, several authors claim that mindfulness not only fosters the control of behavior but also further leads to a self-regulation in terms of stress and anxiety reduction (Brown and Ryan 2003; Schultz and Ryan 2015). Mindfulness stimulates executive control and effective emotional regulations. Thus, it fosters openness and sensitivity to subtle changes in affective states. Additionally, the need for control can be optimized and thereby its execution adapted (Teper et al. 2013). Therefore, mindfulness is beneficial in practical forms of self-regulation, including emotion regulation (Heppner et al. 2015) by enhancing emotional awareness and control (Nielsen and Kaszniak 2006) via cognitive enhancement (Teper et al. 2013).

While many mindfulness studies emphasize effects on mood relevant aspects (Chiesa and Serretti 2009), self-leadership literature focuses more on performance improvements (Neck and Manz 2013). However, even though the strength of self-leadership lies in improving achievement, research shows that the application of self-leadership is also useful to reduce stress (Lucke and Furtner 2015; Neck and Manz 1996). As such, self-leadership is suggested not only as a goal pursuit intervention but also as a means of anticipatory coping (Aspinwall and Taylor 1997; Unsworth and Mason 2012) which might be optimized by conscious attention to and awareness of behavior provided by mindfulness (Brown and Ryan 2003; Levesque and Brown 2007). On the other hand, mindfulness is not only introduced because of its benefits of stress and anxiety reduction (Lynch et al. 2011; Regehr et al. 2013) but also to enhance motivational achievement behavior (Radel et al. 2009), academic self-efficacy (Keye and Pidgeon 2013), and cognitive performance (Lutz et al. 2008; Lynch et al. 2011).

This line of research shows that mindfulness and self-leadership have several theoretical points of contact (e.g., self-regulation, self-observation) and improve similar outcomes such as stress reduction, self-efficacy, and self-determined achievement behavior (Dundas et al. 2016; Lucke and Furtner 2015). Thereby, mindfulness and self-leadership foster attention (Neck and Houghton 2006; Tang et al. 2007) and reduce automatic and impulsive reactions (Bishop et al. 2004) and achievement-oriented behavior regulation, which is crucial in performance improvements (Levesque and Brown 2007; Neck and Houghton 2006). Thus, self-leadership gives students a variety of goal-oriented strategies, for example, behavior-focused strategies, while mindfulness helps to shape or modify implicit tendencies to improve the goal achievement process (Levesque and Brown 2007). The practical application of mindfulness offers exercises on attention, mood, and relaxing (Chiesa and Serretti 2009). On the other hand, the application of self-leadership offers explicit goal pursuit exercises to structure the learning process (Furtner et al. 2012; Neck and Houghton 2006).

In the mindfulness and self-leadership literature, there are a wide range of mostly subjective performance measurements. Furthermore, most of these studies evaluated performance-related outcomes (Norris 2008) and defined performance either as being a cognitive ability (Chiesa et al. 2011) or as higher productivity (Neck and Manz 1996). However, the transfer of these effects to the practical usage and measure of university students’ grades has been of little interest. Research demonstrates that stress is inversely correlated to the grades of students (Struthers et al. 2000). Thus, the relationship between academic stress and academic achievement measured by grades is mediated by the coping style and motivation of college students (Struthers et al. 2000). In order to provide students with an opportunity to develop healthy achievement-oriented self-regulation skills that are applicable to daily life, Tang et al. (2007) suggest to develop an intervention that integrates various methods.

Following the suggestions of Tang et al. (2007), the first aim of the current study is to develop and evaluate an intervention that takes account of both performance-related effects and mental health in the academic domain. In a pilot intervention, we integrated for the first time mindfulness and self-leadership into one practically applicable training named mindfulness-based self-leadership training (MBSLT). The complex combination should provide a stress- and test anxiety-attenuating method to stay abreast of current changes in demanding university tasks. Furthermore, we aim to transfer performance effects to objective measurements. Thus, the second goal of the present study is to evaluate the ecological validity of academic achievement by investigating the effects on grades. It was hypothesized that the successful application of the MBSLT would lead to significantly higher levels of mindfulness and self-leadership in the training group compared to the control group posttreatment. We predicted in our second hypothesis that compared to pretest measures of stress and test anxiety, the intervention group would have significantly lower levels of stress and test anxiety on postintervention measures compared to the waiting list group. We assumed in our third hypothesis that the intervention group would show higher levels of academic self-efficacy than the control group posttreatment. According to previous findings on cognitive performance and enhancement of achievement behavior, we assumed in our fourth hypothesis that the intervention group would achieve significantly better grades than the control group.

## Method

### Participants

A total of 109 bachelor students of the University of Innsbruck participated in the intervention study and took part until the completion of the study. The intervention group consisted of 51 participants (38 women, 13 men; average age *M* = 21.39, SD = 3.08; average study semester *M* = 3.27, SD = 2.35) and the control group of 58 participants (44 women, 14 men; average age *M* = 23.07, SD = 5.43; average study semester *M* = 4.41, SD = 2.90). In order to analyze academic performance, participants were requested to voluntarily give their consent to collecting their grades after the examination period. Thirty-nine participants of the intervention group (30 women, 9 men; average age *M =* 20.90, SD = 2.40; average study semester *M* = 2.67, SD = 1.49) and 41 participants of the control group (30 women, 11 men; average age *M =* 22.83, SD = 3.65; average study semester *M* = 4.34, SD = 2.63) reported their grades. None of the participants indicated suffering from a diagnosed psychiatric disease or having first-degree relatives who did, being under the influence of psychoactive substances or psychopharmacologic treatment. On the examination date, none of the participants had any brain damage or had suffered from severe head injuries in the past (self-report). Informed consent was obtained according to the guidelines of the institutional Ethics Committee.

### Procedure

#### Study Design

The study was designed as a longitudinal randomized controlled trial. In this study, the effects of a 10-week mindfulness-based self-leadership training (MBSLT) on mindfulness, self-leadership, academic achievement, strain, test anxiety, and academic self-efficacy were evaluated by means of a questionnaire-based test. Academic performance was examined using objective measurement criteria, i.e., calculating weighted average of achieved grades.

During the summer term, students of all bachelor programs of the University of Innsbruck were introduced via e-mail to the opportunity to participate in this training voluntarily. Students had the opportunity to sign up for the study by completing a questionnaire, which coexisted as the first measurement point (T1). The questionnaire implemented mindfulness, self-leadership, stress, test anxiety, and academic self-efficacy scales. One hundred nine students participated in the intervention study and contributed until completion either in an intervention group or a waiting list group. As the study’s aim was the examination of the effects on stress prevention and academic achievement in healthy young adults, any brain damages and psychiatric disorders served as screened exclusion criteria. After the first assessment point at T1, participants were randomly assigned to either a training group (MBSLT group) or a control group (waiting list).

During a fixed period of 10 weeks, participants of the MBSLT group received the training, whereas participants of the control group received no training. The control group was informed that groups were divided due to the high attendance and received the training at a later point when the study was finished. After the completion of the training or waiting period, all participants were invited again to participate in a second assessment (T2) by completing the same questionnaires as used in T1. In order to reflect as closely as possible the critical variables, T2 took place during the examination period at the end of the summer term. When the training and the examination period were finished, participants were requested to voluntarily give their consent to collect their grades. All data were analyzed anonymously, and participants gave their written informed consent.

#### Intervention

The MBSLT was designed as group intervention for 10–15 participants. Over a time period of 10 weeks, the training took place once a week for 2 h each. Mindfulness and self-leadership contents were taught by one experienced coach using lectures, group work, and guided exercises. To consolidate the acquired knowledge, the participants were given home exercises and were encouraged to write their training experiences and progress down in a daily logbook. To ensure the practical applicability, all exercises were designed to be easily integrated into daily life. Home exercises and training sessions were discussed in a weekly briefing and debriefing procedure. The development of the MBSLT is based on a combination of established mindfulness elements of the MBSR method (Kabat-Zinn 1994, 2013) and self-leadership training suggestions of Neck and Manz (2013). In total, the training consisted of five main mindfulness modules that were based on each other and extended by several self-leadership strategies (see Table 1).

**Table 1:**
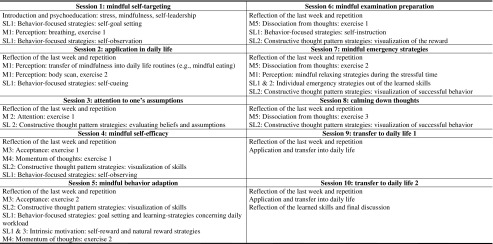
Overview of the mindfulness-based self-leadership training and its single sessions

The numerals (M1–M6; SL1–SL3) refer to the number of each module and its sequential order

*M* mindfulness treatment, *SL* self-leadership treatment

#### MBSLT Mindfulness and Self-Leadership Modules

The first mindfulness module that focused on (M1) perception built up the basic requirement for mindfulness practice and consisted of several basic exercises (e.g., breathing exercises, body scan) in which participants developed a feeling for their body and learned how to integrate mindfulness into their daily lives. In this module, participants learned to keep attention on one experience. The second module expanded the first module and consisted of (M2) attention exercises to develop the ability to intentionally switch from one aspect to another flexibly. The third mindfulness module emphasized the (M3) acceptance of unchangeable events. In this module, participants learned how to deal with failure and daily hassles. The thematic priority of the third mindfulness module was the realization and nonjudgmental comprehension of the (M4) momentum of thoughts. In this phase, participants could discover that the mind consists of a continual coming and going of thoughts. Based on the previous modules, the fifth module consisted of exercises dealing with the (M5) dissociation from thoughts. In this last module, participants learned to detach themselves from undesired thoughts by focusing on the present moment with a nonjudgmental attitude.

Each mindfulness module was combined with a number of self-leadership strategies. Concerning self-leadership, the MBSLT included three primary strategies, with accurately defined subcategories, suggested by Neck and Manz (2013). Regarding (SL1) behavior-focused strategies, the MBSLT consisted self-goal setting, self-reward, self-observation, and self-cueing and reminding elements. The second main self-leadership strategy included (SL2) constructive thought pattern strategies such as visualizing successful performances, self-talk, and evaluating beliefs and assumption. Additionally, the MBSLT comprised the origination, creation, and upholding of motivation by (SL3) using natural reward strategies.

#### MBSLT Sessions

The MBSLT sessions themselves were constructed in a process dynamic way that became more exam-specific the closer the examination period came. Depending on the temporal distance to exams, the sessions concentrated on different aspects. Session 1, “mindful self-targeting,” focused on goal-setting strategies and basic mindfulness exercises on perception and self-observation. In the first step, participants learned the basics of defining and reaching their academic goals in a mindful way.

In session 2, “application in daily life,” participants were taught to remind themselves of their goals and daily mindfulness practice in terms of a real-life training by self-cueing and self-reminding strategies.

Session 3, “attention to one’s assumptions,” dealt with self-reflexive processes. The self-evaluation of constructive beliefs and assumptions was enhanced by attention- and awareness-improving mindfulness exercises. Furthermore, mindfulness exercises to improve relaxation skills of participants were repeated.

Session 4, “mindful self-efficacy,” emphasized participants’ ability to deal with academic setbacks in an accepting and mindful way. Students learned to take an attitude of nonjudgmental comprehension of the momentum of thoughts and understand that thoughts come and go. By visualization, participants improved their self-efficacy concerning their academic goals. Via a daily logbook, participants were able to observe their own daily progress (concerning learning success, stress, and anxiety) in a mindful way. By setting their own weekly learning plan and self-reminders, students learned to structure their examination preparation.

Session 5, “mindful behavior adaption,” built on the self-observation experiences of former sessions. By being aware of their current progress, participants learned to adapt and structure their learning behavior in order to reach their self-set goals. Intrinsic motivation was enhanced by an adequate use of (natural) self-rewarding strategies. To deepen the awareness of reward experiences, former mindfulness exercises were repeated.

The exercises of session 6, “mindful examination preparation,” enabled participants to dissociate themselves from thoughts, for example, stressful thoughts concerning examination failure. Visualization of rewards and successful exams enhanced motivation. Self-instructions helped students to focus and structure examination preparation, in this process, mindfulness facilitated the application of focusing strategies. Again, mindfulness was used to reduce the stress caused by academic stressors. Students learned to conduct mindful relaxing exercises before they started their learning session.

Session 7, “mindful emergency strategies,” dealt with the deepening of dissociation from stressful thoughts and mindful relaxing strategies. With the help of the learned self-leadership and mindfulness skills, participants learned to develop their own “emergency” strategies for examination and pressure situations, for example, to refocus through self-talk, and mindful breathing to calm down. To enhance participants’ self-efficacy successful examination, behavior was visualized.

Session 8, “calming down thoughts,” focused on the use of constructive thought patterns, as this session was close to the stressful examination period. Students identified stressful thoughts concerning their exams. The mindfulness exercises of dissociation from thoughts enhanced the effects, for example, letting thoughts pass, and led to relaxing effects.

Session 9 and 10, “transfer to daily life 1 and 2,” focused on the repetition of self-leadership and mindfulness strategies and skills learned before. Participants learned to transfer the skills into daily life and examination situations.

### Measures

Trait mindfulness was measured with a validated German version (Kobarg 2007) of the 15-item Mindful Attention and Awareness Scale (MAAS, Brown and Ryan 2003). The MAAS has a single-factor structure (consisting of 15 items) focusing on the measurement of the present moment awareness. The scale was designed for assessing people who have no particular former meditation training experience. Answer options to each item follow a six-point Likert scale ranging from 1 “almost always” to 6 “almost never.” A sample item is “I am aware of thoughts I’m having when my mood changes.” In this study, the overall mindfulness scale showed a high internal consistency at T1 (*α* = .89) and at T2 (*α* = .89).

For measurement of self-leadership, the German validated Revised Self-Leadership Questionnaire-Deutsch (RSLQ-D, Andreßen and Konradt 2007), containing 27 items, was implemented. The RSLQ-D is a marginally revised scale of the original scale (Houghton and Neck 2002), containing 27 items. All items are rated on a five-point Likert scale ranging from 1 “I completely disagree” to 5 “I completely agree.” The scale measures nine subscales building up the three self-leadership strategies behavior-focused strategies (e.g., “I use written notes to remind myself of what I need to accomplish”), natural reward strategies (e.g., “I focus my thinking on the pleasant rather than the unpleasant aspects of my job activities”), and constructive thought pattern strategies (e.g., “I think about my own beliefs and assumptions whenever I encounter a difficult situation”). In this study, the overall self-leadership scale demonstrated a high internal consistency at T1 (*α* = .81) and at T2 (*α* = .85).

The current perceived stress was assessed with the 20-item Perceived Stress Questionnaire (PSQ 20, Fliege et al. 2001), a shortened validated German version of the original PSQ (Levenstein et al. 1993). The PSQ 20 consists of the following four subscales (five items each): tension (e.g., “You feel mentally exhausted”), demands (e.g., “You feel that too many demands are being made on you”), worries (e.g., “You fear you may not manage to attain your goal”), and joy (e.g., “You feel you are doing things you really like”). All items are rated on a four-point Likert scale ranging from 1 “almost never” to 4 “usually.” In this study, the overall scale demonstrated a high internal consistency at T1 (*α* = .94) and at T2 (*α* = .93).

For measurement of test anxiety, the German Prüfungsangstfragebogen was used (PAF; Hodapp et al. 2011). The PAF is a revised and shortened version of the German Test Anxiety Inventory (TAI-G, Hodapp et al. 1982) and consists of 20 items. The PAF includes the following four subscales (five items each): emotionality (e.g., “My heart is pounding”), worry (e.g., “I am thinking about the consequences of failing”), lack of confidence (e.g., “I’m confident concerning my own performance”), and interference (e.g., “I’m preoccupied by other thoughts, and thus distracted”). Answer options to each item follow a four-point Likert scale ranging from 1 “almost never” to 4 “almost always.” In this study, the overall scale demonstrated a high internal consistency at T1 (*α* = .89) and at T2 (*α* = .89).

For measurement of academic self-efficacy, the Self-Efficacy Scale of Pintrich and De Groot (1990) was slightly adjusted and translated into German. The Self-Efficacy Scale consists of nine items focusing on perceived competence and confidence in study performance concerning the current semester (e.g., “I am sure that I can do an excellent job on the problems and tasks assigned for this class). The Self-Efficacy Scale has a single-factor structure, and each item is rated on a seven-point Likert scale ranging from 1 “almost always” to 7 “almost never.” In this study, the overall scale showed a high internal consistency at T1 (*α* = .89) and at T2 (*α* = .91).

The objective academic performance was examined using the grade point average (GPA) of the current semester. All grades, that were voluntarily reported back, were weighted with the workload credits (ECTS) depending on the examination subject (European Commission 2016). In Austria, scholastic grades use a five-point grading scale: 1 = excellent, 2 = good, 3 = satisfactory, 4 = adequate, and 5 = unsatisfactory, which is the lowest possible grade and the only failing grade (Federal Ministry of Teaching and Art, Austria 1974).

### Data Analyses

First, we tested whether the two groups differed on any parameter at admission (T1), which indicated that the groups could be considered equivalent. Second, in order to assess differences between groups over time, a mixed ANOVA for each dependent variable of interest was computed with time as within-subject factor (T1, T2) and condition (intervention-control group) as between-subject factor. Third, to assess changes over time within each group, we applied *t* tests for repeated measures on T1 and T2 scores for each group separately. Lastly, to compare the intervention and control group at posttreatment, a between-group *t* test at T2 was used. Degrees of freedom were corrected in case of deviance from sphericity (Greenhouse-Geisser). Effect sizes are reported by partial eta squared *η*_*P*_^2^(.01 = small; .06 = medium; .14 = large) for analyses of variance and by Cohen’s *d* (.30 = small; .50 = medium; .80 = large) (Cohen 1988).

## Results

### Effects on Mindfulness and Self-Leadership

**Fig. 1:**
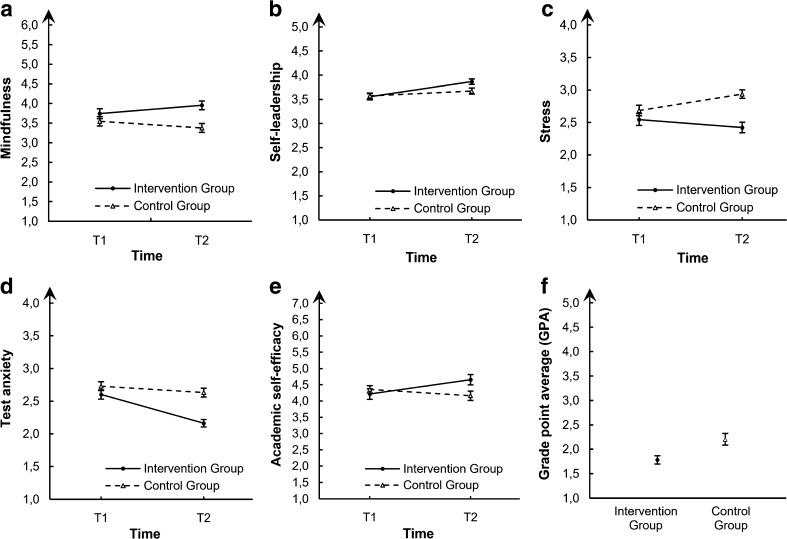
Illustration of developments of **a** mindfulness, **b** self-leadership, **c** stress, **d** test anxiety, **e** academic self-efficacy over time, and **f** academic performance posttreatment. *Error bars* represent standard errors. *T1* first measurement point, *T2* second measurement point

In hypothesis 1, we predicted that the application of MBSLT would positively affect mindfulness scores. First, a *t* test for independent measures on mindfulness scores at T1 showed no differences between groups [*t*(107) = 1.12, *p* = .266]. The repeated ANOVA on mindfulness scores showed a significant group × time interaction [*F*(1,107) = 5.83, *p* = .017, *η*_*P*_^2^ = .05]. We found no main effect for time [*F*(1,107) = .07, *p* = .793], whereas the main effect of group reached a significant level [*F*(1,107) = 6.73, *p* = .011, *η*_*P*_^2^ = .06]. To further substantiate group effects on mindfulness over time, we applied *t* tests for repeated measures for each group separately on scores in T1 and T2 as well as a *t* test for independent measures on scores in T2. Although the former statistical testing revealed that mindfulness did not change over time, neither for the control group [*t*(57) = 1.90, *p* = .063; T1: *M* = 3.55, SD = .91; T2: *M* = 3.38, SD = .88] nor the intervention group [*t*(50) = −1,58, *p* = .121; T1: *M* = 3.74, SD = .90, T2: *M* = 3.95, SD = .80], the latter *t* test on mindfulness scores in T2 showed that participants of the intervention group reported significantly higher mindfulness levels than participants of the control group [*t*(107) = 3.55, *p* = .001, *d* = .68]. Both the group × time interaction effect and the difference between groups in T2 support our predictions in hypothesis 1 (see Fig. 1a).

According to our first hypothesis, we predicted further that the MBSLT would lead to higher levels of self-leadership in the intervention group compared with the control group. First, a *t* test for independent measures on self-leadership scores at T1 showed no differences between the groups [*t*(107) = −.20, *p* = .845]. The observation of self-leadership showed a significant group × time interaction [*F*(1,107) = 8.69, *p* = .004, *η*_*P*_^2^ = .08] indicating differences in the development of self-leadership scores between the two groups over time. The main effect of time [*F*(1,107) = 33.42, *p* < .001, *η*_*P*_^2^ = .24] reached significant levels, whereas there was no main effect of the group factor on self-leadership scores [*F*(1,107) = 1.33, *p* = .252]. To further substantiate group effects on self-leadership over time, we applied *t* tests for repeated measures for each group separately on scores in T1 and T2 as well as a *t* test for independent measures on scores in T2. The former analysis showed that in the control group, self-leadership increased significantly from T1 to T2 [*t*(57) = −2.10, *p* = .04, *d* = .22; T1: *M* = 3.57, SD = 0.44; T2: *M* = 3.67, SD = .46]. Further statistical analyses revealed significant increases of self-leadership in the intervention group [*t*(50) = −5.90, *p* < 0.001, *d* = .73; T1: *M* = 3.55, SD = .45, T2: *M* = 3.87, SD = .40]. These findings indicate that the intervention group showed stronger increases in self-leadership levels than the control group. A paired *t* test at T2 confirmed that participants in the intervention group reached significantly higher self-leadership scores than participants in the control group [*t*(107) = 2.31, *p* = .023, *d* = .44]. The group × time interaction effect and higher self-leadership scores in T2 compared to the control group as well as the increasing self-leadership scores over T1 to T2 are in line with hypothesis 1 (see Fig. 1b).

### Effects on Perceived Stress and Test Anxiety

In our second hypothesis, we predicted that the MBSLT would lead to lower levels of perceived stress in the intervention group compared with the control group. First, a *t* test for independent measures on stress scores at T1 showed no differences between groups [*t*(107) = −1.14, *p* = .258]. The conducted ANOVA revealed a significant time × group interaction [*F*(1,107) = 12.11, *p* = .001, *η*_*P*_^2^ = 0.10] and a significant main effect of group [*F*(1,107) = 10.82, *p* = .001, *η*_*P*_^2^ = .09]. There was no main effect of the time factor [*F*(1,107) = 1.41, *p* = .237]. To study group effects on stress over time more closely, we applied *t* tests for repeated measures for each group separately on scores in T1 and T2 as well as a *t* test for independent measures on scores in T2. The former statistical testing revealed that stress levels in the control group significantly increased from T1 to T2 [*t*(57) = −3.71, *p* < 0.001, *d* = 0.45, T1: *M* = 2.68, SD = .62, T2: *M* = 2.94, SD = .50], whereas the intervention group showed no significant changes in stress levels [*t*(50) = 1.45, *p* = .154, T1: *M* = 2.55, SD = .65, T2: *M* = 2.42, SD = .58], indicating that stress levels of the intervention group remained stable from before training and after training, i.e., during the examination interval. Confirming these findings, participants of the control group reported significantly higher levels of stress than the intervention group at posttreatment measurement (T2) [*t*(107) = −4.97, *p* < .001, *d* = .95]. Both the group × time interaction effect and the difference between groups in T2 support our predictions in hypothesis two (see Fig. 1c).

Hypothesis 2 predicted further that the MBSLT would positively affect test anxiety levels. To ensure independent measures on test anxiety on T1, we conducted a *t* test in a first step. We found no differences between groups [*t*(107) = −1.30, *p* = .196] in T1, indicating that both groups could be treated equally. For the further analyses of test anxiety, we conducted a repeated ANOVA showing a significant main effect of time [*F*(1,107) = 30.31, *p* < .001, *η*_*P*_^2^ = .22], which indicates that overall mean levels of test anxiety changed from T1 to T2. Further, the repeated ANOVA showed a significant time × group interaction [*F*(1,107) = 12.32, *p* = .001, *η*_*P*_^2^ = .10] and a significant main effect of group [*F*(1,107) = 14.15, *p* < .001, *η*_*P*_^2^ = .12]. In a next step, we analyzed group effects on mindfulness over time in detail. The *t* tests for repeated measures indicate that test anxiety levels in the intervention group significantly lowered from T1 to T2 [*t*(50) = 6.53, *p* < .001, *d* = .96; T1: *M* = 2.60, SD = .50, T2: *M* = 2.16, SD = .41], whereas the control group showed no changes in test anxiety levels [*t*(57) = 1.40, *p* = .168, T1: *M* = 2.73, SD = .51; T2: *M* = 2.63, SD = .51]. A between-group *t* test at T2 indicates a strong reduction of test anxiety through MBSLT, showing a lower level of test anxiety in trained participants than in the nontrained participants [*t*(107) = −5.29, *p* < .001, *d* = 1.06]. The group × time interaction effect, lower test anxiety scores in T2 compared to the control group, and the decrease of test anxiety scores over T1 to T2 in the intervention group confirm our predictions of hypothesis 2 (see Fig. 1d).

### Effects on Academic Self-Efficacy

Our third hypothesis predicted better academic self-efficacy levels in the MBSLT group compared with the control group posttreatment. First, a *t* test for independent measures on academic self-efficacy scores at T1 showed no differences between groups [*t*(107) = −.74, *p* = .459]. As expected, the conducted repeated ANOVA concerning academic self-efficacy displayed significant time × group interactions [*F*(1,107) = 11.78, *p* = .001, *η*_*P*_^2^ = .01]. Effects of time [*F*(1,107) = 1.73, *p* = .191] and group [*F*(1,107) = .90, *p* = .344] were not significant. To further analyze group effects on self-efficacy over time, we conducted *t* tests for repeated measures for each group separately on scores in T1 and T2 as well as a *t* test for independent measures on scores in T2. The former analysis showed that in the intervention group, academic self-efficacy increased significantly from T1 to T2 [*t*(50) = −2.88, *p* = .006, *d* = .54, T1: *M* = 4.21, SD = .16, T2: *M* = 4.66, SD = .16], whereas self-efficacy levels in the control group did not change from T1 to T2 [*t*(57) = 1.78, *p* = .081, T1: *M* = 4.36, SD = 0.87, T2: *M* = 4.16, SD = 1.09]. Further posttreatment analysis at T2 confirmed these results, revealing significantly higher academic self-efficacy levels in the intervention group than in the control group [*t*(107) = 2.28, *p* = .025, *d* = .44]. The group × time interaction effect, higher academic self-efficacy scores in T2 compared to the control group, and the increase of academic self-efficacy scores over T1 to T2 in the intervention group confirm our predictions of hypothesis 3 (see Fig. 1e).

### Assessment of Academic Performance

In our main hypothesis, we assumed that students who participated in the intervention group would have better grades (GPA) at the end of the semester, i.e., during the examination period, compared to students in the control group. Thirty-nine participants of the MBSLT group and 41 participants in the control group returned their grades via their recent semester report. On average, students in the intervention group took *M* = 4.49 (SD = 1.52) exams, whereas students of the control group took *M* = 3.80 (SD = 2.00) exams. First, in order to compare both groups, the mean grades were calculated based on the single grades weighted with the workload credits (ECTS) of each exam (MBSLT group: *M* = 14.00 ECTS, SD = 5.28; control group: *M* = 13.05 ECTS, SD = 7.45). Descriptive data showed an average GPA of *M* = 1.78 (SD = 0.53) in the intervention group and an average GPA of *M* = 2.20 (SD = .77) in the control group. To further expatiate on differences concerning academic performance in the intervention and control group, we conducted a between-group *t* test in T2. This showed that participants of the intervention group had better grades than participants of the control group [*t*(70.942) = −2.85, *p* = .006, *d* = .63]. In line with our main hypothesis, this result indicates that the MBSLT led to higher academic performance (see Fig. 1f).

## Discussion

This study demonstrates significant improvements in academic performance by integrating both mindfulness and self-leadership into one training. The MBSLT was successfully applied in the academic context as a pilot intervention. As expected in our main hypothesis, the MBSLT group showed significantly better GPA compared to its control group. In line with our hypotheses, the MBSLT influenced mindfulness, self-leadership, stress, test anxiety, self-efficacy, and performance positively. The findings are consistent with previous studies that suggest using self-regulative interventions to assist students to manage stress and anxiety, and to cope with academic achievement experiences (Furtner et al. 2012; Ratanasiripong et al. 2015). However, this study explicitly investigates the effects of a mixture of stress and achievement-oriented strategies on both mental health-related variables as well as academic performance (GPA). We aimed to discuss the effects of the MBSLT with other interventions. Thus, in order to enhance the comparability of our results, we conducted a post hoc effect size comparison with studies that used similar outcomes but applied only a mindfulness-based or a self-leadership intervention.

In accordance with previous research, we found significant effects on mindfulness (e.g., Gallego et al. 2014; Zenner et al. 2014) and self-leadership (Furtner et al. 2012). Previous studies suggest that mindfulness-based interventions have strongest effects on the mindfulness subscales observing and nonreactivity (Ramler et al. 2016). These effects might be optimized by the MBSLT suggesting a combination of several self-leadership and mindfulness exercises that might enhance quality and the effects of self-observing processes (Furtner et al. 2017). Unfortunately, it is not possible to determine which is the most significant element of the MBSLT in academic settings, which should be examined in further studies.

Concerning self-leadership, the MBSLT revealed higher self-leadership levels in the intervention group, promoting goal setting and achievement-orientated behavior (Lucke and Furtner 2015; Neck and Manz 2013). There is only one self-leadership training in the academic setting (Furtner et al. 2012), which showed similar effect sizes as the MBSLT on self-leadership but did not examine stress or achievement using objective ratings. The contribution of the MBSLT is that one single training can lead to significant effects on both mindfulness and self-leadership. Thus, the MBSLT was able to replicate the effects of separated mindfulness and self-leadership interventions (Furtner et al. 2012; Zenner et al. 2014). An interesting finding was that although mindfulness did not increase through the intervention over time, at the postintervention measurement, both groups differed in mindfulness. In fact, we could show that in the control group, mindfulness showed tendencies to decrease, whereas feelings of stress increased (Cunha and Paiva 2012; Lynch et al. 2011). These findings indicate that the MBSLT enhanced self-leadership and further enabled participants to keep their mindfulness level even though overall stress naturally increased due to the stressful examination period (Furtner et al. 2017). In support of our first hypothesis, we can show that the MBSLT was effectively applied in the academic context.

The MBSLT showed changes in stress and test anxiety levels over time on a moderate level and exposed large effect sizes after the intervention. These effect sizes are in accordance with meta-analysis of mindfulness-based interventions for healthy people that found large effects on stress levels and moderate effects on anxiety changes (Chiesa and Serretti 2009; Khoury et al. 2015). However, a recent meta-analysis shows that compared to other populations, students only benefit from generalized mindfulness training to a small or moderate level, indicating that students might have special needs (Khoury et al. 2015). Accordingly, Ramler et al. (2016) found large effects on cortisol levels but could not enhance academic adjustment by an isolated mindfulness-based training. In contrast, the MBSLT gives students both general mindfulness exercises and a variety of self-leadership skills to structure examination preparation, for example, goal setting and self-reminding. The MBSLT takes into account that the amount of academic stress peaks at the end of the semester (Regehr et al. 2013) and therefore gives students several strategies dependent upon process dynamics. Thereby, the exercises became more domain-specific the closer the stressful examination period came (e.g., session seven “mindful emergency strategies”). In fact, while the level of stress increased in the control group, the MBSLT group was able to hold their stress level stable over time. The MBSLT creates an attenuating effect on naturally increasing stress and test anxiety levels during academic high stress examination periods by supporting students with specific individual tools that are adaptive for specific pressure peaks. Accordingly, after the intervention, the MBSLT group showed large effects on lower test anxiety and stress compared to the control group even though stress levels became naturally higher (Regehr et al. 2013).

The large impact on test anxiety levels is in line with effect sizes of a mindfulness-based intervention on evaluation anxiety of Dundas et al. (2016). Studies that evaluated nonspecific anxiety reached effect sizes from no effect (Gouda et al. 2016) to low (Rosenzweig et al. 2003) up to large effects (Barbosa et al. 2013), indicating that the MBSLT reaches good effects compared to single mindfulness-based interventions, but in addition focused on achievement outcomes (Bamber and Kraenzle Schneider 2016). Furthermore, so far, there is no self-leadership training that evaluates its impact on anxiety or focuses on mood-relevant outcomes in the academic context. However, self-leadership intervention studies in the occupational context suggest several practical strategies that can be adapted for academic domain-specific strains and could be further examined in the MBSLT approach (Lucke and Furtner 2015). The investigated separate self-leadership trainings so far revealed small to moderate effects on strain and stress, but have not been conducted in the academic context yet (Lucke and Furtner 2015; Unsworth and Mason 2012).

According to the transactional model of stress, the MBSLT might enable participants to influence how the stressor relates to them, even when the demanding environment is not changeable (Lazarus and Folkman 1984), by its enhancement of acceptance (Kabat-Zinn 1994), self-regulative coping with stressful events (Bishop et al. 2004; Chiesa and Serretti 2009), and better executive control (Teper et al. 2013). Beyond this, the MBSLT with the help of its behavioral self-leadership strategies might provide students with additional psychological resources to prevent future stressors from occuring (Unsworth and Mason 2012). These effects are very promising and should be further examined using longer evaluation periods and follow-up measurements after 6 and 12 months. In line with this research and our second hypothesis, our findings suggest that trained students were able to develop a successful way of coping with academically demanding stress situations and were able to keep their perceived stress level low and reduce test anxiety.

In accordance with our third hypothesis, results showed an improvement of academic self-efficacy, through the MBSLT. While the self-efficacy in the control group decreased, self-efficacy in the intervention group increased over time. Consequently, during the stressful examination period, the intervention group had higher levels of academic self-efficacy compared to its control group. Remarkably, we could find these effects despite the second measurement point that took place during the approaching examination period (Regehr et al. 2013). Furthermore, previous research shows that test anxiety and stress scores are negatively associated with academic self-efficacy (Howell and Buro 2011; Zajacova et al. 2005). Accordingly, the MBSLT might promote academic self-efficacy by its attenuating effect on stress and test anxiety. These findings indicate that students felt more confident through the intervention and learned to cope with stressful academic events, which predicts academic performance (Unsworth and Mason 2012). Concerning the effects of other separate mindfulness interventions on self-efficacy, previous results are inconsistent: There are former studies that showed larger effect sizes on academic self-efficacy levels than the MBSLT (Dundas et al. 2016), while others showed the same (Gouda et al. 2016), lower or no effects on general self-efficacy (Charoensukmongkol 2014; Phang et al. 2015), but could not enhance anxiety, mood, or mindfulness (Gockel et al. 2013; Gouda et al. 2016) with their intervention. Based on that comparison, we suggest the use of the MBSLT as intervention to enhance not only academic self-efficacy alone but also holistic mood-relevant aspects and performance. An approach to achieve large effect sizes in self-efficacy changes might be to enhance training intensity and conduct the MBSLT for a longer period of time, which needs further analysis.

In accordance with our fourth hypothesis, the MBSLT group had significant better grades compared to its control group (Hall 1999; Lucke and Furtner 2015). The MBSLT gives students a tool to focus consistently on academic goals by self-goal setting, visualization, and self-observation and suggests cognitive as well as behavior focused strategies to structure examination preparation (Neck and Manz 2013). On the other hand, it might increase students’ ability to stay focused in learning situations (Hjeltnes et al. 2015) and foster the cognitive performance by encouraging better concentration, strengthening the ability to focus (Bishop et al. 2004; Valentine and Sweet 1999) and affecting memory during exams (Zenner et al. 2014).

The wide range of instruments used to measure performance in former studies requires a careful examination of data. In a meta-analysis of MBSR interventions in schools, Felver et al. (2016) pointed out that no study included objective data on student educational outcomes such as grades and declared it as major critical point of most academic mindfulness interventions. Hall (1999) found moderate effects of a mindfulness-based intervention on academic grades but conducted a time-intensive training over one full semester. Similarly, but not in the academic setting, a self-leadership intervention led to small effects on examination grades of soldiers (Lucke and Furtner 2015). Compared to these studies, we found moderate effects of the 10-week MBSLT on grades, but in contrast to other studies invented a time-saving training that was mainly devised for an easy day-to-day use in the academic setting. The objective performance criterion (GPA) increased ecological validity of the introduced intervention in academic context and proved the successful practical transfer to relevant aspects of academic achievement. However, in order to make the MBSLT more comparable to other academic interventions, further research on mindfulness and self-leadership trainings and its effects on grades is needed.

Uniquely different from previous research, the MBSLT takes into account both mood-relevant aspects and improvements in objective performance criteria (here GPA). Compared to other isolated mindfulness or self-leadership interventions, the MBSLT showed the same or even better effects in terms of stress, test anxiety, self-efficacy, and objective performance enhancement within the same time span of training (Chiesa et al. 2011; Lucke and Furtner 2015). An interesting finding of post hoc tests was that the MBSLT group was able to hold their stress and mindfulness level stable over time, while mindfulness and stress resilience decreased in the control group. These results indicate that the intervention had a stress stabilization effect during naturally higher stress periods, which is important for students to perform well in exams (Khoury et al. 2015; Ramler et al. 2016). Further research needs to clarify the role of the MBSLT in its maintaining of mindfulness and stress stabilizing function during the semester.

Within the MBSLT, the combination of mindfulness and self-leadership offers a variety of day-to-day strategies. Thereby, the effects of self-leadership strategies might be enhanced by mindfulness, as mindful people act more consciously and evaluate situations carefully in order to become goal-oriented in this situation (Furtner et al. 2017; Radel et al. 2009). Furthermore, mindful people could use several self-leadership strategies (e.g., visualization, self-rewarding) more effectively as they think about what they are going to do in present situations. Therefore, rewarding aspects of desired behavior (natural rewarding self-leadership strategies) might be better perceived with a mindful attitude (Brown and Ryan 2003). Mindfulness might support the selection process of self-leadership strategies as it reduces impulsive and automatic behavior (Bishop et al. 2004; Furtner et al. 2017) and enhances the achievement of intrinsic goals and aspirations in a self-determined way (Deci et al. 2015). This indicates that mindfulness has the potential to moderate the effects of self-leadership on several outcomes within the MBSLT. Additionally, the self-leadership approach within the MBSLT gives practical guidelines on how to develop goals step by step and remind oneself of them in an effective way (e.g., self-cueing, visualization of goals) (Neck and Manz 2013). Within the MBSLT, self-leadership offers explicit practical goal pursuit exercises to structure the learning process. On the other hand, mindfulness offers practical exercises to foster attention and relaxing. Because of the specific combination, both mindfulness and self-leadership exercises mutually supplement each other within the MBSLT in the academic context. Thus, the training of both constructs within one intervention supplements the strength of each method. Moreover, effects of self-leadership should be enhanced by the moderating function of mindfulness.

From our findings, we see the potential of the newly developed MBSLT and its combination of mindfulness and self-leadership in addressing a variety of outcomes by one single training that is applicable in daily academic life. The MBSLT gives students a wide variety of practical tools to enhance their personal efficacy in exam preparation (e.g., structure through self-goal setting and self-cuing) and during exams (e.g., mindful breathing to relax and self-instructions to focus) (Kabat-Zinn 2013; Neck and Manz 2013). Thus, the MBSLT focusses on a process dynamic application but gives advice for general mood improvements as well. Furthermore, it might have the potential to prevent stressors from arising (Unsworth and Mason 2012). Mindfulness might enhance the effects of self-leadership on promoting performance as it improves self-regulation functioning (Furtner et al. 2017; Levesque and Brown 2007) and self-leadership helps to structure the learning process. This new combination and its potential should be further investigated in terms of how mindfulness and self-leadership interplay and which strategies of the MBSLT are crucial either in mood or performance improvements.

## Limitations and Directions for Future Research

This study shows the effects on stress and test anxiety levels, as well as on academic self-efficacy and performance. Nevertheless, there are some points that have to be discussed critically: First, even though the pilot study examines the great potential in the academic context, results are limited, as we are not able to examine whether effects originated from mindfulness, self-leadership, or the combination of both. We propose that mindfulness might enhance the effects of self-leadership on different outcomes; however, the mechanisms on how both constructs interact and their additive effects remain only theoretical. As such, we are not able to determine if mindfulness and self-leadership or the combination of both is the most significant factor in reducing stress, test anxiety, and improving self-efficacy or grades. Therefore, we suggest for further studies to examine the holistic effects of the MBSLT compared to an isolated mindfulness and self-leadership intervention and a control group using similar outcomes.

Second, the generalizability concerning the average student population may be limited, as students who participated voluntarily were probably specifically attracted to stress prevention and achievement-related tasks. However, this setting ensured a high level of ecological validity as the training was conducted in a real-world situation during the semester and university students with a subjectively perceived need could join the training.

Third, to meet students’ needs, the intervention was constructed to be easily incorporated into an average student’s life. The randomized design prevented the possibility that bias might be introduced by differences in starting values between intervention and control group. The random allocation to either an intervention or a control group enabled us to compare the effects of our intervention to those of a waiting list group which increased the expressiveness of our findings. Particularly, the use of an objective criterion for measuring the academic performance as an effect of the executed MBSLT is a distinctive feature of the present study. Nevertheless, because of a limited amount of studies that focused explicitly on the same outcomes (grades, stress, test anxiety, self-efficacy, mindfulness, and self-leadership), comparability is restricted. As such, our study only provides a relative comparison to other mindfulness and self-leadership interventions. It would be desirable for further studies to examine the effects on academic grades in order to compare the MBSLT with studies that applied only a self-leadership or mindfulness-based intervention.

Fourth, in order to analyze the supposed mindfulness maintaining and stress-attenuating function of the MBSLT, we would recommend a further examination of the effects of the MBSLT in a context where external stressors and ascendancies remain mainly stable over the study period. Even though the study revealed great effects on several outcomes over time, the study design does not allow us to make any statements about medium- and long-term effects. Our training was time-efficient and economical; however, effects on outcomes might be improved by conducting the MBSLT for a longer period of time. To analyze longitudinal effects of how the MBSLT might prevent future stressors from arising, follow-up measures after 6 and 12 months on stress and grades should be explored for further research. Finally, we suggest transferring the promising findings of the present study to a broader context to assess the effects of the mindfulness-based self-leadership training in different domains (e.g., clinical setting, military, management, industry, sports).

The results clearly demonstrate the positive outcomes of the new combination of mindfulness and self-leadership: enabling participants to promote both academic performance and psychological states in the same time span as former interventions that separated mindfulness and self-leadership (Khoury et al. 2015; Lucke and Furtner 2015). It is remarkable that the positive effects on academic performance could be confirmed with objective data by comparing the GPA (Hall 1999), demonstrating that the MBSLT is a high potential training as a tool to succeed within high-stress academic environments. The new intervention gives advice on how to integrate the practice in daily life in order to improve a broad variety of outcomes with only one intervention. As students seem to need a specific intervention, the MBSLT is sensitive to process dynamics and stress peaks (Regehr et al. 2013; Tang et al. 2007). The MBSLT is a promising approach concerning the combination of mindfulness and self-leadership in real-life educational pressure settings. The MBSLT is ready to be applied, further examined, and verified in different contexts.

## References

[CR1] Alexander CN, Langer EJ, Newman RI, Chandler HM, Davies JL (1989). Transcendental meditation, mindfulness, and longevity: an experimental study with the elderly. Journal of Personality and Social Psychology.

[CR2] Andreßen P, Konradt U (2007). Messung von Selbstführung: Psychometrische Überprüfung der deutschsprachigen Version des Revised Self-Leadership Questionnaire. Zeitschrift für Personalpsychologie.

[CR3] Ang RP, Huan VS (2006). Relationship between academic stress and suicidal ideation: testing for depression as a mediator using multiple regression. Child Psychiatry and Human Development.

[CR4] Aspinwall LG, Taylor SE (1992). Modeling cognitive adaptation: a longitudinal investigation of the impact of individual differences and coping on college adjustment and performance. Journal of Personality and Social Psychology.

[CR5] Aspinwall LG, Taylor SE (1997). A stitch in time: self-regulation and proactive coping. Psychological Bulletin.

[CR6] Bamber MD, Kraenzle Schneider J (2016). Mindfulness-based meditation to decrease stress and anxiety in college students: a narrative synthesis of the research. Educational Research Review.

[CR7] Barbosa P, Raymond G, Zlotnick C, Wilk J, Toomey R, Mitchell J (2013). Mindfulness-based stress reduction training is associated with greater empathy and reduced anxiety for graduate healthcare students. Education for Health.

[CR8] Baumeister RF, Vohs KD (2007). Self-regulation, ego depletion, and motivation. Social and Personality Psychology Compass.

[CR9] Baumeister RF, Heatherton TF, Tice DM (1994). Losing control: how and why people fail at self-regulation.

[CR10] Bewick B, Koutsopoulou G, Miles J, Slaa E, Barkham M (2010). Changes in undergraduate students’ psychological well-being as they progress through university. Studies in Higher Education.

[CR11] Bishop S, Lau M, Shapiro SL, Carlson L, Anderson M, Carmody J, Devins G (2004). Mindfulness: a proposed operational definition. Science and Practice.

[CR12] Brown KW, Ryan RM (2003). The benefits of being present: mindfulness and its role in psychological well-being. Journal of Personality and Pocial Psychology.

[CR13] Brown KW, Ryan RM, Creswell JD (2007). Mindfulness: theoretical foundations and evidence for its salutary effects. Psychological Inquiry.

[CR14] Carver CS, Scheier MF (1981). Attention and self-regulation: a control theory approach to human behavior.

[CR15] Carver CS, Scheier MF (1994). Situational coping and coping dispositions in a stressful transaction. Journal of Personality and Pocial psychology.

[CR16] Carver CS, Scheier MF (2001). On the self-regulation of behavior.

[CR17] Charoensukmongkol P (2014). Benefits of mindfulness meditation on emotional intelligence, general self-efficacy, and perceived stress: evidence from Thailand. Journal of Spirituality in Mental Health.

[CR18] Chiesa A, Serretti A (2009). Mindfulness-based stress reduction for stress management in healthy people: a review and meta-analysis. Journal of Alternative and Complementary Medicine.

[CR19] Chiesa A, Calati R, Serretti A (2011). Does mindfulness training improve cognitive abilities? A systematic review of neuropsychological findings. Clinical Psychology Review.

[CR20] Cohen, J. (1988). *Statistical power analysis for the behavioral sciences*. Hillsdale, NJ: Erlbaum.

[CR21] Cunha M, Paiva MJ (2012). The test anxiety in adolescents: the role of self-criticism and acceptance and mindfulness skills. The Spanish Journal of Psychology.

[CR22] Deci EL, Ryan RM, Schultz PP, Niemiec CP, Brown KW, Creswell JD, Ryan RM (2015). Being aware and functioning fully: mindfulness and interest-taking within self-determination theory. Handbook of mindfulness: theory, research, and practice.

[CR23] Dundas I, Thorsheim T, Hjeltnes A, Binder PE (2016). Mindfulness based stress reduction for academic evaluation anxiety: a naturalistic longitudinal study. Journal of College Student Psychotherapy.

[CR24] European Commission. (2016). *ECTS Users’ Guide*. http://ec.europa.eu/education/ects/ects_en.htm. Accessed 6 Sept 2016

[CR25] Federal Ministry of Teaching and Art Austria. (1974). *Bundesgesetzblatt für die Republik Österreich*. https://www.ris.bka.gv.at/GeltendeFassung.wxe?Abfrage=Bundesnormen&Gesetzesnummer=10009375. Accessed 8 Sep 2016.

[CR26] Felver JC, Celis-de Hoyos CE, Tezanos K, Singh NN (2016). A systematic review of mindfulness-based interventions for youth in school settings. Mindfulness.

[CR27] Fliege H, Rose M, Arck P, Levenstein S, Klapp BF (2001). Validierung des “Perceived Stress Questionnaire” (PSQ) an einer deutschen Stichprobe. Diagnostica.

[CR28] Furtner MR, Sachse P, Exenberger S (2012). Learn to influence yourself: full range self-leadership training. Journal of the Indian Academy of Applied Psychology.

[CR29] Furtner MR, Rauthmann JF, Sachse P (2015). Unique self-leadership: a bifactor model approach. Leadership.

[CR30] Furtner, M.R., Tutzer, L., & Sachse, P. (2017). The mindful self-leader: investigating relations between self-leadership and mindfulness. *Social Behavior and Personality* (in press).

[CR31] Gallego J, Aguilar-Parra JM, Cangas AJ, Langer ÁI, Mañas I (2014). Effect of a mindfulness program on stress, anxiety and depression in university students. The Spanish Journal of Psychology.

[CR32] Gockel A, Burton D, James S, Bryer E (2013). Introducing mindfulness as a self-care and clinical training strategy for beginning social work students. Mindfulness.

[CR33] Gouda S, Luong MT, Schmidt S, Bauer J (2016). Students and teachers benefit from mindfulness-based stress reduction in a school-embedded pilot study. Frontiers in Psychology.

[CR34] Hall PD (1999). The effect of meditation on the academic performance of african american college students. Journal of Black Studies.

[CR35] Heppner WL, Spears CA, Vidrine JI, Wetter DW, Ostafin BD, Robinson MD, Meier BP (2015). Mindfulness and emotion regulation. Handbook of mindfulness and self-regulation.

[CR36] Hjeltnes A, Binder P-E, Moltu C, Dundas I (2015). Facing the fear of failure: an explorative qualitative study of client experiences in a mindfulness-based stress reduction program for university students with academic evaluation anxiety. International journal of qualitative studies on health and well-being.

[CR37] Hodapp V, Laux L, Spielberger C (1982). Theorie und Messung der emotionalen und kognitiven Komponente der Prüfungsangst. Zeitschrift für Differentielle und Diagnostische Psychologie.

[CR38] Hodapp V, Rohrmann S, Ringeisen T (2011). Prüfungsangstfragebogen: PAF.

[CR39] Hofmann W, Schmeichel B, Baddeley A (2012). Executive functions and self-regulation. Trends in Cognitive Sciences.

[CR40] Houghton JD, Neck CP (2002). The revised self-leadership questionnaire. Journal of Managerial Psychology.

[CR41] Howell AJ, Buro K (2011). Relations among mindfulness, achievement-related self-regulation, and achievement emotions. Journal of Happiness Studies.

[CR42] Howell AJ, Digdon NL, Buro K, Sheptycki AR (2008). Relations among mindfulness, well-being, and sleep. Personality and Individual Differences.

[CR43] Kabat-Zinn J (1990). Full catastrophe living: the program of the stress reduction clinic at the University of Massachusetts Medical Center.

[CR44] Kabat-Zinn J (1994). Wherever you go, there you are. Mindfulness meditation in every day life.

[CR45] Kabat-Zinn J (2013). Gesund durch Meditation: Das große Buch der Selbstheilung mit MBSR.

[CR46] Keng S-L, Smoski MJ, Robins CJ (2011). Effects of mindfulness on psychological health: a review of empirical studies. Clinical Psychology Review.

[CR47] Keye MD, Pidgeon AM (2013). An investigation of the relationship between resilience, mindfulness, and academic self-efficacy. Open Journal of Social Sciences.

[CR48] Khoury B, Sharma M, Rush SE, Fournier C (2015). Mindfulness-based stress reduction for healthy individuals: a meta-analysis. Journal of Psychosomatic Research.

[CR49] Kobarg A (2007). Deutsche Adaptation der Mindfulness Attention Awareness Scale (MAAS) Validierung am Gesundheitsstatus und Gesundheitsverhalten.

[CR50] Konradt U, Andreßen P, Ellwart T (2009). Self-leadership in organizational teams: a multilevel analysis of moderators and mediators. European Journal of Work and Organizational Psychology.

[CR51] Lazarus R, Folkman S (1984). Stress, appraisal, and coping.

[CR52] Levenstein S, Prantera C, Varvo V, Scribano ML, Berto E, Luzi C, Andreoli A (1993). Development of the perceived stress questionnaire: a new tool for psychosomatic research. Journal of Psychosomatic Research.

[CR53] Levesque C, Brown KW (2007). Mindfulness as a moderator of the effect of implicit motivational self-concept on day-to-day behavioral motivation. Motivation and Emotion.

[CR54] Locke EA, Latham GP (1990). A theory of goal setting and task performance.

[CR55] Locke EA, Latham GP (2002). Building a practically useful theory of goal setting and task motivation. A 35-year odyssey. The American Psychologist.

[CR56] Lucke GA, Furtner MR (2015). Soldiers lead themselves to more success: a self-leadership intervention study. Military Psychology.

[CR57] Lutz A, Slagter HA, Dunne JD, Davidson RJ (2008). Attention regulation and monitoring in meditation. Trends in Cognitive Sciences.

[CR58] Lynch S, Gander ML, Kohls N, Kudielka B, Walach H (2011). Mindfulness-based coping with university life: a non-randomized wait-list-controlled pilot evaluation. Stress and Health.

[CR59] MacKenzie MJ, Baumeister RF, Ostafin BD, Robinson MD, Meier BP (2015). Self-regulatory strength and mindfulness. Handbook of mindfulness and self-regulation.

[CR60] Manz CC (1986). Self-leadership: toward an expanded theory of self-influence processes in organizations. Academy of Management Review.

[CR61] Manz CC (1991). Leading employees to be self-managing and beyond: toward the establishment of self-leadership in organizations. Journal of management systems.

[CR62] Manz CC, Sims HP (1980). Self-management as a substitute for leadership: a social learning theory perspective. The Academy of Management Review.

[CR63] McKean KJ (1994). Academic helplessness: applying learned helplessness theory to undergraduates who give up when faced with academic setbacks. College Student Journal.

[CR64] Mrazek MD, Franklin MS, Phillips DT, Baird B, Schooler JW (2013). Mindfulness training improves working memory capacity and GRE performance while reducing mind wandering. Psychological Science.

[CR65] Neck CP, Houghton JD (2006). Two decades of self-leadership theory and research. Journal of Managerial Psychology.

[CR66] Neck CP, Manz CC (1996). Thought self-leadership: the impact of mental strategies training on employee cognition, behavior, and affect. Journal of Organizational Behavior.

[CR67] Neck CP, Manz CC (2013). Mastering self-leadership: empowering yourself for personal excellence.

[CR68] Nielsen L, Kaszniak AW (2006). Awareness of subtle emotional feelings: a comparison of long-term meditators and nonmeditators. Emotion.

[CR69] Norris SE (2008). An examination of self-leadership. Emerging Leadership Journeys.

[CR70] Phang C, Mukhtar F, Ibrahim N, Keng S, Mohd S, Mohd Sidik S (2015). Effects of a brief mindfulness-based intervention program for stress management among medical students: the mindful-gym randomized controlled study. Advances in Health Sciences Education.

[CR71] Pintrich PR, De Groot EV (1990). Motivational and self-regulated learning components of classroom academic performance. Journal of Educational Psychology.

[CR72] Prussia GE, Anderson JS, Manz CC (1998). Self-leadership and performance outcomes: the mediating influence of self-efficacy. Journal of Organizational Behavior.

[CR73] Radel R, Sarrazin P, Legrain P, Gobancé L (2009). Subliminal priming of motivational orientation in educational settings: effect on academic performance moderated by mindfulness. Journal of Research in Personality.

[CR74] Ramler TR, Tennison LR, Lynch J, Murphy P (2016). Mindfulness and the college transition: the efficacy of an adapted mindfulness-based stress reduction intervention in fostering adjustment among first-year students. Mindfulness.

[CR75] Ratanasiripong, P., Park, J. F., Ratanasiripong, N., & Kathalae, D. (2015). Stress and anxiety management in nursing students: biofeedback and mindfulness meditation. *Journal of Nursing Education, 54*(9), 520–524. 10.3928/01484834-20150814-07 26334339

[CR76] Regehr C, Glancy D, Pitts A (2013). Interventions to reduce stress in university students: a review and meta-analysis. Journal of Affective Disorders.

[CR77] Rosenzweig S, Reibel DK, Greeson JM, Brainard GC, Hojat M (2003). Mindfulness-based stress reduction lowers psychological distress in medical students. Teaching and Learning in Medicine.

[CR78] Ryan RM, Deci EL, Greenberg J, Sander KL, Pyszczynski T (2004). Autonomy is no illusion: self-determination theory and the empirical study of authenticity, awareness, and will. Handbook of experimental existential psychology.

[CR79] Schultz PP, Ryan RM, Ostafin BD, Robinson MD, Meier BP (2015). The “why,” “what,” and “how” of healthy self-regulation: mindfulness and well-being from a self-determination theory perspective. Handbook of mindfulness and self-regulation.

[CR80] Shapiro SL, Schwartz GE, Bonner G (1998). Effects of mindfulness-based stress reduction on medical and premedical students. Journal of Behavioral Medicine.

[CR81] Shapiro SL, Carlson LE, Astin JA, Freedman B (2006). Mechanisms of mindfulness. Journal of Clinical Psychology.

[CR82] Stewart GL, Carson KP, Cardy RL (1996). The joint effects of conscientiousness and self-leadership training on employee self-directed behavior in a service setting. Personnel Psychology.

[CR83] Stewart S, Lam T, Betson C, Wong M, Wong A (1999). A prospective analysis of stress and academic performance in the first two years of medical school. Medical Education.

[CR84] Struthers CW, Perry RP, Menec VH (2000). Anexamination of the relationship among academic stress, coping, motivation, and performance in college. Research in Higher Education.

[CR85] Tang Y-Y, Ma Y, Wang J, Fan Y, Feng S, Lu Q (2007). Short-term meditation training improves attention and self-regulation. Proceedings of the National Academy of Sciences of the United States of America.

[CR86] Teper R, Segal ZV, Inzlicht M (2013). Inside the mindful mind: how mindfulness enhances emotion regulation through improvements in executive control. Current Directions in Psychological Science.

[CR87] Unsworth K, Mason C (2012). Help yourself: the mechanisms through which a self-leadership intervention influences strain. Journal of Occupational Health Psychology.

[CR88] Valentine ER, Sweet PLG (1999). Meditation and attention: a comparison of the effects of concentrative and mindfulness meditation on sustained attention. Mental Health, Religion & Culture.

[CR89] Zajacova A, Lynch SM, Espenshade TJ (2005). Self-efficacy, stress, and academic success in college. Research in Higher Education.

[CR90] Zenner C, Herrnleben-Kurz S, Walach H (2014). Mindfulness-based interventions in schools: a systematic review and meta-analysis. Frontiers in Psychology.

